# Home measures against low air humidity which may alleviate health problems

**DOI:** 10.31744/einstein_journal/2021AO5484

**Published:** 2021-06-17

**Authors:** Larissa Pereira Guerra, Larissa Martins Vieira de Andrade, Daiany Caroline Joner, Daniel Strozzi

**Affiliations:** 1 Pontifícia Universidade Católica de Goiás GoiâniaGO Brazil Pontifícia Universidade Católica de Goiás , Goiânia , GO , Brazil .; 2 Universidade Federal de Goiás GoiâniaGO Brazil Universidade Federal de Goiás , Goiânia , GO , Brazil .

**Keywords:** Communicable diseases, Climate change, Air pollution, Humidifiers, Wet towel

## Abstract

**Objective:**

Humidity and temperature are fundamental for the balance in the life cycle of living beings and, consequently, for maintaining the well-being of the human population and reducing the prevalence of infectious diseases. Thus, in order to mitigate the impact of climate change, especially in the period when humidity is not the ideal, it is necessary to adopt some assistance measures. The present experimental study aims to elucidate what would be the recommended option to improve the quality of life of the human being and to clarify which resources (air humidifier, bucket of water or wet towel) will be effective to improve the humidity of the air in times of drought and low moisture.

**Methods:**

The experimental study was carried out with INKBIRD hygrometers allowing the analysis of the variation of air humidity throughout the day. Three forms of treatment were established: humidifier, wet towel and bucket of water. In each room, two hygrometers were placed equidistant from the occupant of the room and their respective treatment that varied between 1m and 2m away from the headboard indoor each room. In addition, two environments were used as controls, one being an external environment and the other an internal closed environment, totaling five rooms for the study. The rooms were monitored between the end of July and the end of August 2019 in Goiania (GO).

**Results:**

Although assistance measures are used to significantly improve air pollution in times of extreme drought, there was a significant difference between them. The humidifier and a wet towel had 7.50% and 5.71% more humidity in the external relation (external control), respectively, more efficient. The volume of water, however, did not show significant difference (p>0.05) and, therefore, there was no variation.

**Conclusion:**

The humidifier and the towel are treatments considered more efficient, and that there was a significant effect of distance on humidity. Therefore, 1m of distance is more efficient in increasing and/or maintaining air humidity, inducing improvements in the populations’ health.

## INTRODUCTION

Humidity and temperature are abiotic factors that influence the climate and they are fundamental to the balance of the life cycle of living beings. However, urbanization has generated changes in climate profile and, consequently, it has aggravated or facilitated the emergence of diseases to the human population. To investigate the effects of climate change on health there is need to measure how these factors vary over time to allow the creation of measures that can mitigate health-related negative effects. ^( 1 - 4 )^

These effects range from the interaction between air humidity and temperature to air pollution, thermal sensation, and precipitation, which are factors responsible for regulating the metabolism of organisms. In the Center-West region of Brazil, the state of Goiás, on the months of July and August, the impact of climate change is already observed. Currently, these months present longer drought and low humidity that they used to be, and this change exacerbates health problems. ^( 5 - 7 )^

Humidity makes the atmosphere dense and prevents the dissipation of pollutants, bacteria, and viruses that easily survive in the period of low humidity. The excessive emission of pollutants has caused several direct damage to public health such as worsening of respiratory problems, respiratory, ocular and dermatological allergies, asthma, headaches, dryness of the upper airways that may lead to nosebleeds, dry and irritated throats, a feeling of sand in the eyes that become hyperemic, skin dryness, and fatigue. These health problems are worse when the air humidity is not at the ideal level recommended by the World Health Organization (WHO), which should be around 40% to 70%. Besides this ideal level of humidity, there is also a classification of the criticality states of low air humidity, according to a psychrometric scale: humidity between 21% and 30% is considered an attention state; between 12% and 20% an alert; and below 12% an emergency. ^( 2 , 8 - 11 )^

While the humidity is below the ideal to adopt care measures whenever possible is necessary to minimize health- related problems. These measures involve the use of an air humidifier, bucket with water, and wet towel. However, little is known whether these resources have significant effectiveness in reducing the impacts caused by low air humidity, nor what would be the ideal location of these devices within rooms.

Air humidity is a worldwide issue especially in times of drought. Low humidity influences the well-being of the population, affecting growth, development, and quality of life. These unfavorable relative humidity levels increase the prevalence of infectious diseases that may compromise the population and makes pathogens more contagious. This study aims to elucidate what would be the recommended resources and what should be the distance of them from the individual to enhance population welfare in times of drought and low humidity.

## OBJECTIVE

To determine which resources are effective to improve the air humidity in dry seasons and indicate the best distance for these resources to be from the individual in order to obtain the appropriate level of air humidity, and improve the quality of life of human being.

## METHODS

### Design of the study

The study was carried out in the city of Goiania, state of Goiás, Brazil. To evaluate the most effective treatment to regulate air humidity, we analyzed air humidifier, bucket with water, and wet towel in three identical 15m ^2^ rooms at the same house using similar furniture accommodations at the indoor environment of a residential building. The INKBIRD hygrometers were attached to these accommodations to allow the analysis of the air humidity variation throughout the day. The humidifier used was the G-Tech ultrasonic humidifier, with a cloth wick system to take water from the reservoir, which made the air absorb humidity, and held 3L of water. The towel was 100% cotton and measured 70cmx140cm, manufactured by Karsten. The size of a bath towel and it was able to wet, on average, with 1L of water. The bucket used contained 5L of water.

In each room, two hygrometers were placed distant from the occupant of the room and its respective treatment, varying between 1m and 2m away from the head of the bed indoor each room. In addition, two rooms were used as controls, one outdoor (near the garage) and the other indoors, totalizing five rooms for the study. The rooms were monitored from the end of July to the end of August 2019.

### Statistical analysis

The data collected in percentages of humidity in each room were divided into four periods: dawn, morning, afternoon and evening. Subsequently, average humidity values were calculated for each period. The times established to perform the calculations were within a 2-hour interval, from 1:45 am until 11:45 pm. The times set for the calculations for the dawn period were 1:45 am, 3:45 am, and 5:45 am; for the morning 7:45 am, 9:45 am, and 11:45 am; for the afternoon 1:45 pm, 3:45 pm, and 5:45 pm; for the night 7:45 pm, 9:45 pm, and 11:45 pm.

Repeated measures analysis of variance (ANOVA) was determined to evaluate the difference between the treatments used (humidifier, wet towel, and bucket of water). In this way, we considered as similar the spatial and temporal dependence collected in the five rooms. However, the pre-analysis with this test indicated a collinearity between the monitored factors: period of the day (dawn, morning, afternoon and evening) and treatment type (humidifier, wet towel, water bucket, indoor area, and outdoor area).

A linear mixed model was built, using humidity as the response variable, and treatment type and day period as predictor variables, which considered the fixed factors of the mixed model (see formula below). The room was considered as random factor of the analysis as having specific characteristics in each of the spaces, which could influence the observed humidity. In addition, the autocorrelation value was considered, since the experiment was repeated in the same rooms throughout the study.

$$\begin{array}{c} \text{Model = humidity}∼{\text{Treatment}}^{*}\text{Period,}\\ \;\text{random}=∼1|\text{Room} \end{array}$$

Finally, ANOVA was performed to assess whether there was an effect in terms of distance of the two most efficient treatments on air humidity quality. The treatments ranged from 1m to 2m distance from the bed within each room. The analyses were performed on R platform version 3.6.1 (2019), which is developed by R Development Core Team and freely available at http://www.r-project.org. This software functions as a language in which the codes used for the statistical analyses are open and the user can adapt them as needed.

## RESULTS

**Figure 1:**
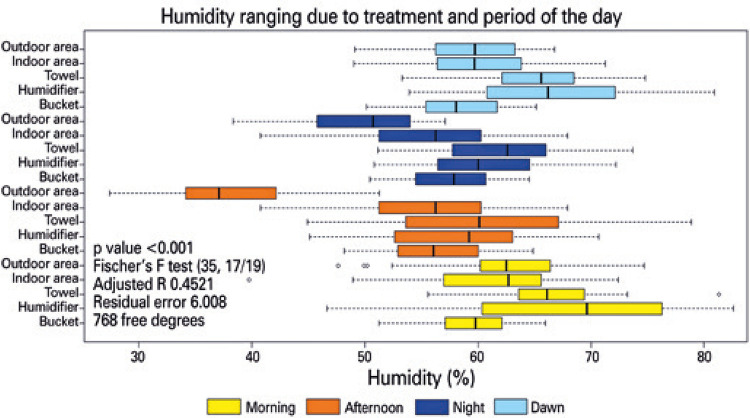
Distribution of observed humidity data obtained between July and August 2019. Each observation is divided into quartiles with the first vertical bar to the left of the rectangle representing the lower limit of the data meaning 25% of the data; in the beginning of the rectangle, the bar cutting through the rectangle represents the measure and thus 50% of the data. The end of the rectangle indicates 75% of the encompassed data, and the vertical bar to the right of the rectangle indicates the upper limit of the data. Points beyond the vertical bars, both to the right and left of the rectangle indicate highly discrepant values (outliers) within the observed values

In general, we observed that efficacy of treatments and periods of the days ranged. The lowest value of humidity (27.40%) was observed in the outdoor in afternoon, whereas the highest value of humidity (82.60%) ( Figure 1 ). Measurements conducted at dawn and morning showed similar values of humidity.

Estimations from the mixed linear model, *i.e* ., the predicted values, indicated a mean humidity value of 59.35% with a standard error of 1.44% ( Table 1 ). When considering the types of treatment, the humidifier had 7.50% more humidity compared with the outdoor area (external control) where no treatment was used. The towel had 5.71% more humidity than the outdoor area. The external and indoor areas and the water bucket, despite having different average results (% estimation), did not differ significantly (p>0.05), and no variation was observed.

**Table 1:** Estimated results by the mixed model of the percentage of humidity

Variables of interest	Estimation (%)	Standard error	Degrees of freedom	*t*	p value
Estimated mean of humidity*	59,359.45	1,447926	765	40,99619	<0.001
Type of treatment					
Indoor area	0,49164	1,972449	3	0,24925	0.8193
Bucket	-1,14567	1,73018	3	-0,66217	0.5552
Towel*	5,71607	1,700881	3	3,36065	<0.05
Humidifier*	7,50219	1,893019	3	3,96308	<0.05
Period of the day					
Morning*	2,7748	1,023283	765	2,71166	<0.01
Night*	-9,47327	1,035618	765	-9,14745	<0.001
Afternoon*	-21,32808	1,174819	765	-18,1544	<0.001
Interaction between treatment and time of the day					
Indoor area/Morning	-2,28859	1,392474	765	-1,64354	0.1007
Bucket/Morning	-1,51569	1,221903	765	-1,24044	0.2152
Towel/Morning	-1,30347	1,200768	765	-1,08553	0.278
Humidifier/Morning	-1,84721	1,341647	765	-1,37682	0.169
Indoor area/Afternoon*	17,34471	1,598366	765	10,85153	<0.001
Bucket/Afternoon*	19,25042	1,402671	765	13,72412	<0.001
Towel/Afternoon*	16,69053	1,378318	765	12,10935	<0.001
Humidifier/Afternoon*	12,70819	1,541154	765	8,24589	<0.001
Indoor Area/Night*	5,5857	1,408104	765	3,96682	<0.05
Bucket/Night*	8,80441	1,235974	765	7,12346	<0.001
Towel/Night*	6,49969	1,21426	765	5,3528	<0.01
Humidifier/Night*	3,12167	1,360835	765	2,29394	<0.05

* Significant result.

All day periods differed significantly (p<0.05), and the afternoon was the period that recorded the lowest average of humidity (about 21.32% less when compared with dawn).

**Figure 2:**
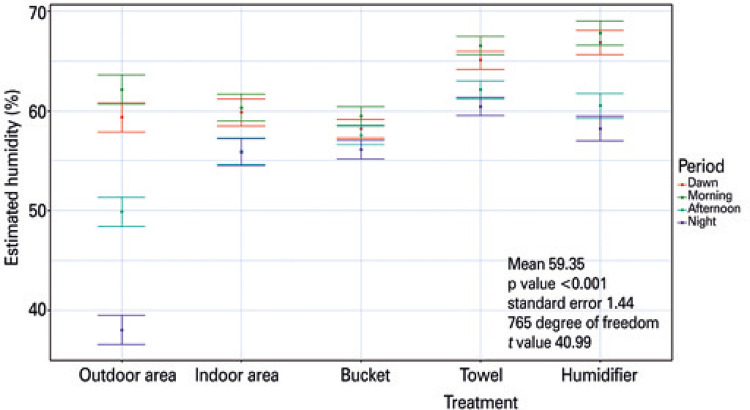
Estimated interaction between treatment type (bucket, towel, and humidifier) and day period (dawn, morning, afternoon, evening). Each color reprents one of the periods analyzed during the experiment. The horizontal bars, lower and upper, indicate the minimum and maximum values of percentage of humidity, while the dot represents the average value of humidity for each type of treatment

The outdoor area, where there was no treatment and, possibly the environment most exposed to evapotranspiration, was the one that differed most between the periods of the day ( Figure 2 ). The variation between the periods was lower when using the bucket with water. The results estimated higher humidity values with the use of the humidifier during the early morning and early at morning hours. This is important because, depending on the functionality of the room (for sleeping, for example), the use of the humidifier may be the most indicated one.

**Figure 3:**
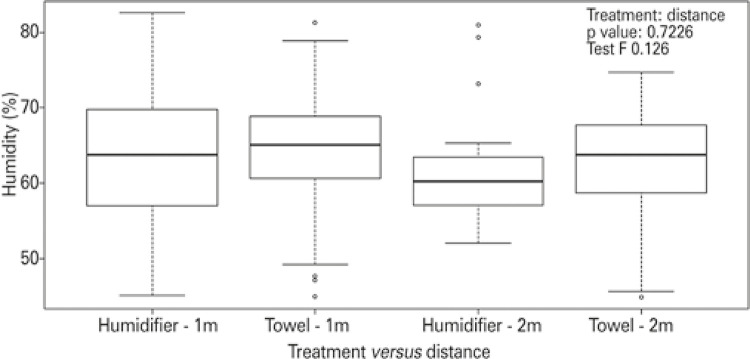
Distribution of observed data about humidity obtained between July and August 2019, considering the treatment used (humidifier and towel) and the distance from the treatment to the bed in the room (1m and 2m). Each observation is divided into quartiles, with the first vertical bar to the left of the rectangle being the lower limit of the data, meaning 25% of them. At the beginning of the rectangle the bar that cuts the rectangle represents the measurement, *i.e* ., 50% of the data. The end of the rectangle indicates 75% of the data encompassed, and the vertical bar to the right of the rectangle indicates the upper limit of the data. Points beyond the vertical bars both to the right and left of the rectangle indicate highly discrepant values (outliers) within the observed values

As the humidifier and the towel were considered the most efficient treatments, the analysis of variance found no difference between treatments ( Figure 3 ), but there was a significant effect of distance on humidity (gl=1; F=4.663; p=0.0315). The distance of 1m was more efficient in increasing and/or maintaining air humidity.

## DISCUSSION

Hygrometers were chosen for the experimental study because they are able to provide an accurate monitoring of temperature and humidity conditions, keep in memory the records of the samples measured throughout the days even with a significant distance between the device and the cell phone that is connected to it via Bluetooth, therefore, guaranteeing high sample specificity. Air humidity is a variable that influences in a multifactorial way the balance of the ecosystem and the organism of all living beings, so its analysis would be more complex. ^( 12 )^ The technical safety of several care measures is questioned because it is not known for sure which measure is really more effective to be exposed in headlines and magazines aimed at the general population in order to inform what really works.

New studies, which evaluate which adaptive measures are really more effective and necessary, because the quality of life and the health of the population are two aspects of the inter-relationship between the city and the environment that are rarely addressed. The human being is the fundamental - but forgotten - point of the environmental issue in large metropolises. An effective feedback on low-cost methods to improve the living conditions of the population in times of drought is of major importance. ^( 5 )^

The experiment, besides proving that the humidifier performs its function of increasing air humidity with mastery also demonstrated that the wet towel is an efficient treatment, and can bring these two statements back to the population, since there was no significant difference between these treatments, according to the analysis of variance. The bucket with water did not impact to the point of making the humidity more suitable for population welfare.

The ideal resources would be the humidifier or the wet towel. However, there are basic precautions to be taken regarding the humidifier. This is due to the accumulation of humidity and dust that occurs in air-conditioned environments, and therefore the proliferation of microbes and bacteria is much higher than in open environments. Suitable measures should be taken in relation to the humidifier, since it is not recommended to leave it on in the room all night long, because without sunlight the air humidity rises naturally, and the excess humidity promoted by the device becomes a problem, culminating in the appearance of mold and mildew. Among the main symptoms of people occupying these environments are infections, allergic and irritating reactions, headaches and joint pains, irritation in the eyes, nose and throat, dry cough, dermatitis, fatigue, drowsiness, difficulty to concentrate, sensitivity to odors, congestion, sinusitis, shortness of breath, allergic rhinitis, bronchial asthma, among others symptoms that are even more increased in dry season. The ideal would be to leave it on in the bedroom for 3 to 4 hours straight and turn it off before going to bed. However, one wonders how it would be possible for the small increase in humidity generated by the humidifier to cause these problems, since the humidity still remains at a level below that recommended by the WHO. Another observation regarding the use of the humidifier would be the need to wash it after each use, in order to prevent it from becoming a focus for fungi and other microorganisms that will be released into the air. ^( 8 , 13 - 15 )^

The evident limitation of studies about which welfare measures are effective and their impact on the population’s life is notorious. However, through previous knowledge and the sum provided by data collection according to the hygrometers used, it was possible to analyze what really works and what is effective. We also identified two possible welfare measures that are very effective for the population, and can make it possible to better construct the strategy of environmental education and prevention methods for the population in times of drought and low humidity, therefore, reducing the impact of diseases.

## CONCLUSION

This study presented the results of a comparative evaluation of the performance of three support measures to promote humidification of the indoor environment of a residential building in a low humidity season between July and August 2019 in city of Goiânia, Goiás, Brazil. The humidifier and the towel were the treatments considered the best measures, and there was a significant effect of distance on humidity. The most efficient distance in increasing and/or maintaining air humidity was 1m. The use of wet towels is environmentally sustainable and economically feasible. This can improve sleep nights for lower to middle class population even in the low humidity and dry conditions in hot environments such as the Brazilian Midwest.
