# Supplementary material for: Home measures against low air humidity which may alleviate health problems
Source: Einstein (Sao Paulo). 2021 Jun 17;19:eAO5484. doi: 10.31744/einstein_journal/2021AO5484 (PMC8225259; doi:10.31744/einstein_journal/2021AO5484)
Supplement: Translation

# Translation: Medidas caseiras contra baixa umidade do ar amenizam agravos na saúde

**Authors:** Larissa Pereira Guerra, Larissa Martins Vieira de Andrade, Daiany Caroline Joner, Daniel Strozzi

**Affiliations:** 1 Brasil Pontifícia Universidade Católica de Goiás, Goiânia, GO, Brasil .; 2 Brasil Universidade Federal de Goiás, Goiânia, GO, Brasil .

**Keywords:** Doenças transmissíveis, Mudança climática, Poluição do ar, Umidificadores, Toalha molhada

## Abstract

**Objetivo:**

A umidade e a temperatura são fundamentais para o equilíbrio no ciclo da vida dos seres vivos e, consequentemente, para manter o bem-estar da população humana e diminuir a prevalência de doenças infecciosas. Visando mitigar o impacto das alterações climáticas, principalmente no período em que a umidade não é a ideal, é preciso adotar algumas medidas assistencialistas. O presente estudo visa elucidar qual seria a opção mais indicada para melhorar a qualidade de vida do ser humano e esclarecer qual melhor recurso (umidificador de ar, balde com água ou toalha molhada) é eficaz para melhorar a umidade do ar em épocas de seca e baixa umidade.

**Métodos:**

Estudo experimental realizado com higrômetros INKBIRD que permitiram a análise da variação da umidade do ar ao longo do dia. Foram estabelecidas três formas de tratamento: umidificador, toalha molhada e balde com água. Em cada quarto, foram colocados dois higrômetros equidistantes do ocupante do quarto e seu respectivo tratamento, que variava entre 1m e 2m de distância da cabeceira da cama dentro de cada cômodo. Além disso, dois ambientes foram utilizados como controle, sendo um externo e outro fechado interno, totalizando cinco cômodos para o estudo. Os cômodos foram monitorados entre o final do mês de julho até final do mês de agosto de 2019 em Goiânia (GO).

**Resultados:**

Apesar de as medidas assistencialistas serem utilizadas para melhora significativa da umidade do ar em épocas de extrema seca, há uma diferença significativa entre elas. O umidificador e a toalha molhada possuíram 7,50% e 5,71% a mais de umidade em relação à área externa (controle externo), respectivamente, sendo considerados mais eficientes. Já o balde de água não se diferenciou significativamente (p>0,05), não havendo variação.

**Conclusão:**

O umidificador e a toalha foram os tratamentos considerados mais eficientes, com efeito significativo da distância sobre a umidade. Portanto, 1m de distância é mais eficiente no aumento e/ou na manutenção da umidade do ar, induzindo melhorias na saúde da população.

## INTRODUÇÃO

A umidade e a temperatura são fatores abióticos que compõem o clima e fundamentais para o equilíbrio no ciclo de vida dos seres vivos. Entretanto, a urbanização tem gerado mudanças no perfil climático e, consequentemente, agravado ou facilitado o surgimento de doenças na população humana. Para investigar os efeitos das mudanças climáticas sobre a saúde, é importante mensurar como esses fatores variam ao longo do tempo e, assim, permitir a criação de medidas que podem mitigar tais efeitos negativos.^( 1 - 4 )^

Esses efeitos vão desde a interação entre a umidade do ar e a temperatura sobre a poluição atmosférica, a sensação térmica e a precipitação, as quais são responsáveis por regular o metabolismo dos organismos. Na Região Centro-Oeste do Brasil, estado de Goiás, nos meses de julho e agosto, já é possível perceber o impacto das alterações climáticas. Esse são meses de seca e baixa umidade, o que agrava os problemas na saúde.^( 5 - 7 )^

A umidade torna a atmosfera densa, não deixando ocorrer a dissipação dos poluentes, das bactérias e dos vírus, que, no período de baixa umidade, sobrevivem com facilidade. A emissão excessiva de poluentes tem causado vários prejuízos diretos para a saúde pública, como piora dos problemas respiratórios, das alergias respiratórias, oculares e dermatológicas, asma, dores de cabeça e ressecamento de vias aéreas superiores, levando a sangramento nasal, garganta seca e irritada, sensação de areia nos olhos que ficam hiperemiados, ressecamentos de pele e fadiga. Esses problemas de saúde se agravam quando a umidade do ar não está no nível ideal recomendado pela Organização Mundial da Saúde (OMS), o qual deve ser em torno de 40% a 70%. Além desse nível ideal de umidade, também há uma classificação dos estados de criticidade da baixa umidade do ar, de acordo com uma escala psicrométrica: umidade entre 21% e 30% é considerada estado de atenção; entre 12% e 20%, alerta e abaixo de 12%, emergência.^( 2 , 8 - 11 )^

Enquanto a umidade está abaixo do ideal, é possível e necessária a adoção de medidas assistencialistas, com o objetivo de minimizar os agravos à saúde. Tais medidas envolvem o uso de umidificador de ar, balde com água e toalha molhada. No entanto, não sabemos qual desses recursos possui uma eficácia significante, a fim de reduzir os impactos causados pela baixa umidade do ar, e nem a localização ideal desses dispositivos dentro dos cômodos.

Sabe-se que a umidade do ar é um problema de questão mundial, principalmente em épocas de seca. A baixa umidade causa efeito no bem-estar da população, afetando o crescimento, o desenvolvimento e a qualidade de vida. Além disso, esses níveis desfavoráveis de umidade relativa aumentam a prevalência de doenças infecciosas, comprometendo a população e tornando os patógenos mais contagiosos. Assim, este trabalho visa elucidar qual seria a opção recomendada e qual deve ser a distância do recurso ao indivíduo para melhorar o bem-estar populacional em épocas de seca e baixa umidade.

## OBJETIVO

Esclarecer quais recursos são eficazes para melhorar a umidade do ar em época de seca e indicar a melhor distância que esses recursos devem ficar do indivíduo, visando obter o nível da umidade do ar adequado para melhorar a qualidade de vida do ser humano.

## MÉTODOS

### Delineamento amostral

O estudo foi realização no município de Goiânia, estado de Goiás, Brasil. Para avaliar qual o tratamento mais eficaz na regulação da umidade do ar, foram analisados três quartos idênticos de 15m^2^na mesma casa com acomodações semelhantes dos móveis no ambiente interno de uma edificação residencial, no Bairro Jardins Milão. Os higrômetros INKBIRD foram fixados nessas acomodações, permitindo a análise da variação da umidade do ar ao longo do dia.

Foram estabelecidas três formas de tratamento: umidificador, toalha molhada e balde com água. O umidificador utilizado foi o da marca G-Tech ultrassônico, com sistema de pavio de pano para tirar água do reservatório, que fazia com que o ar absorvesse a umidade, e comportava 3L de água. A toalha era 100% de algodão, da marca Karsten. Media 70cmx140cm, correspondendo ao tamanho de uma toalha de banho, e era molhada, em média, com 1L de água. O balde continha 5L de água.

Em cada quarto, foram colocados dois higrômetros equidistantes do ocupante do quarto e de seu respectivo tratamento, variando entre 1m e 2m de distância da cabeceira da cama dentro de cada cômodo. Além disso, dois ambientes foram utilizados como controle, sendo um externo (próximo à garagem) e outro fechado interno, totalizando cinco cômodos para o estudo. Os cômodos foram monitorados entre o final do mês de julho até final do mês de agosto de 2019.

### Análise estatística

Os dados coletados em percentagem de umidade, em cada cômodo, foram divididos em quatro períodos: madrugada, manhã, tarde e noite. Foram, então, calculados valores médios de umidade para cada período. Os horários estabelecidos para realizar os cálculos foram com intervalo de 2 horas, a partir de 1h45 até 23h45. Os horários determinados para os cálculos no período da madrugada foram 1h45, 3h45 e 5h45; pela manhã foram: 7h45, 9h45 e 11h45; pela tarde: 13h45, 15h45 e 17h45, e 19h45, 21h45 e 23h45 pela noite.

Foi determinada a realização da análise de variância (ANOVA) de medidas repetidas para avaliar a diferença entre os tratamentos utilizados (umidificador, toalha molhada e balde de água). Dessa forma, assumiu-se a dependência espacial e temporal coletada nos cinco cômodos. Entretanto, a pré-análise com esse teste indicou a colinearidade entre os fatores monitorados: período do dia (madrugada, manhã, tarde e noite) e tipo tratamento (umidificador, toalha molhada, balde de água, área interna e área externa).

Foi construído um modelo linear misto, utilizando como variável resposta a umidade, e tipo de tratamento e período do dia como variáveis preditoras, sendo considerados os fatores fixos do modelo misto (ver fórmula a seguir). O cômodo foi considerado o fator aleatório da análise, por ter características particulares em cada um dos espaços, as quais poderiam influenciar na umidade observada. Além disso, foi considerado o valor de autocorrelação, já que o experimento foi repetido nos mesmos cômodos ao longo do estudo.

$$\begin{array}{c} \text{Modelo}=\text{umidade}∼\text{Tratamento* Período,}\\ \text{random}=∼1|\text{Cômodo} \end{array}$$

Por último, foi realizada a ANOVA para avaliar se havia efeito da distância dos dois tratamentos mais eficientes na qualidade da umidade do ar. Os tratamentos variaram entre 1m e 2m de distância da cama dentro de cada cômodo. As análises foram realizadas em plataforma R versão 3.6.1 (2019), desenvolvida pelo *R Development Core Team* e disponível gratuitamente em http://www.r-project.org. Esse software funciona como uma linguagem, na qual os códigos utilizados para as análises estatísticas são abertos e o usuário pode adaptá-los conforme sua necessidade.

## RESULTADOS

De forma geral, houve variação dos tratamentos, bem como nos períodos do dia. O menor valor de umidade (27,40%) foi observado na área externa no período da tarde, enquanto o maior valor (82,60%) foi observado utilizando o umidificador no período da manhã ( Figura 1 ). Os períodos da madrugada e da manhã possuíram valores mais semelhantes de umidade.

**Figura 1:** Distribuição dos dados observados de umidade obtidos entre julho e agosto de 2019. Cada observação está dividida em quartis, sendo a primeira barra vertical à esquerda do retângulo o limite inferior dos dados, englobando 25% dos dados; o início do retângulo, a barra que corta o retângulo representam a medida e, assim, 50% dos dados. O final do retângulo indica 75% dos dados englobados, e a barra vertical à direita do retângulo indica o limite superior dos dados. Os pontos além das barras verticais, tanto à direita quanto à esquerda do retângulo, indicam valores muito discrepantes ( *outliers* ) dentro dos valores observados

As estimativas do modelo linear misto, ou seja, os valores preditos, indicaram um valor médio de umidade de 59,35%, com erro padrão de 1,44% ( Tabela 1 ). Ao considerar os tipos de tratamento, o umidificador teve 7,50% a mais de umidade em relação à área externa (controle externo), onde nenhum tratamento foi utilizado. A toalha teve 5,71% a mais de umidade que a área externa. As áreas externa e interna e o balde de água, apesar de possuírem médias diferentes (estimativa %), não se diferenciam significativamente (p>0,05), não havendo variação.

**Tabela 1:** Resultados estimados pelo modelo misto da percentagem de umidade

Variáveis de interesse	Estimativa (%)	Erro padrão	Graus de liberdade	*t*	Valor de p
Média de umidade estimada*	59.359.45	1.447926	765	40.99619	<0,001
Tipo de tratamento					
Área interna	0.49164	1.972449	3	0.24925	0,8193
Balde	-1.14567	1.73018	3	-0.66217	0,5552
Toalha*	5.71607	1.700881	3	3.36065	<0,05
Umidificador*	7.50219	1.893019	3	3.96308	<0,05
Período do dia					
Manhã*	2.7748	1.023283	765	2.71166	<0,01
Noite*	-9.47327	1.035618	765	-9.14745	<0,001
Tarde*	-21.32808	1.174819	765	-18.1544	<0,001
Interação tratamento e período do dia					
Área interna/manhã	-2.28859	1.392474	765	-1.64354	0,1007
Balde/manhã	-1.51569	1.221903	765	-1.24044	0,2152
Toalha/manhã	-1.30347	1.200768	765	-1.08553	0,278
Umidificador/manhã	-1.84721	1.341647	765	-1.37682	0,169
Área interna/tarde*	17.34471	1.598366	765	10.85153	<0,001
Balde/tarde*	19.25042	1.402671	765	13.72412	<0,001
Toalha/tarde*	16.69053	1.378318	765	12.10935	<0,001
Umidificador/tarde*	12.70819	1.541154	765	8.24589	<0,001
Área interna/noite*	5.5857	1.408104	765	3.96682	<0,05
Balde/noite*	8.80441	1.235974	765	7.12346	<0,001
Toalha/noite*	6.49969	1.21426	765	5.3528	<0,01
Umidificador/noite*	3.12167	1.360835	765	2.29394	<0,05

* Resultados significativos.

Todos os períodos do dia se diferenciaram significativamente (p<0,05), sendo o período da tarde aquele que registrou menor média de umidade (cerca de 21,32% a menos quando comparado com a madrugada).

A área externa, onde não houve nenhum tipo de tratamento e, possivelmente, o ambiente mais exposto a evapotranspiração, foi a que mais se diferenciou entre os períodos do dia ( Figura 2 ). Já a variação entre os períodos foi menor ao utilizar o balde com água. Os resultados estimaram valores mais altos de umidade com uso do umidificador durante a madrugada e a manhã. Isso é importante, pois, a depender da funcionalidade do quarto (para dormir, por exemplo), o uso do umidificador pode ser o mais indicado.

**Figura 2:** Estimativa de interação entre tipo de tratamento (balde, toalha e umidificador) e período do dia (madrugada, manhã, tarde, noite). Cada cor expressa um dos períodos analisados durante o experimento. As barras horizontais, inferior e superior, indicam os valores mínimos e máximos de percentagem de umidade, enquanto o ponto representa o valor médio de umidade para cada tipo de tratamento

Sendo o umidificador e a toalha os tratamentos considerados mais eficientes, a análise de variância não constatou diferença entre os tratamentos ( Figura 3 ), porém houve efeito significativo da distância sobre a umidade (gl=1; F=4.663; p=0,0315). A distância de 1m foi mais eficiente no aumento e/ou manutenção da umidade do ar.

**Figura 3:** Distribuição dos dados observados acerca da umidade, obtidos entre julho e agosto de 2019, considerando o tratamento utilizado (umidificador e toalha) e a distância do tratamento à cama no cômodo (1m e 2m). Cada observação está dividida em quartis, sendo a primeira barra vertical à esquerda do retângulo o limite inferior dos dados, englobando 25% deles, o início do retângulo, a barra que corta o retângulo representa a medida e, desse modo, 50% dos dados. O final do retângulo indica 75% dos dados englobados, e a barra vertical à direita do retângulo indica o limite superior dos dados. Os pontos além das barras verticais, tanto à direita quanto à esquerda do retângulo, indicam valores muito discrepantes ( *outliers* ) dentro dos valores observados

## DISCUSSÃO

A escolha dos higrômetros para o estudo experimental se deu por ele ser capaz de fornecer um monitoramento preciso das condições de temperatura e umidade, mantendo na memória os registros das amostras medidas ao longo dos dias, mesmo com distância significativa entre o aparelho e o celular que está conectado com ele via *bluetooth* , assegurando, assim, alta especificidade da amostra. A umidade do ar é uma variável que influencia de maneira multifatorial no equilíbrio do ecossistema e do organismo de todos os seres vivos, logo sua análise seria mais complexa.^( 12 )^A segurança técnica de diversas medidas assistencialistas é questionada, pois não se sabe ao certo qual medida é realmente mais eficaz para ser exposta em manchetes e revistas voltadas para a população em geral, a fim de informar o que realmente funciona.

Novos estudos, que avaliem quais medidas adaptativas são realmente mais eficazes, são necessários, pois a qualidade de vida e a saúde da população são dois aspectos da inter-relação entre cidade e meio ambiente raramente abordados, sendo o ser humano o ponto fundamental – mas esquecido da questão ambiental nas grandes metrópoles. É de extrema importância uma devolutiva eficaz sobre métodos de baixo custo para melhorar as condições de vida da população em épocas de secas.^( 5 )^

O experimento, além de comprovar que o umidificador exerce sua função de aumentar a umidade do ar com maestria, também demonstrou que a toalha molhada é um tratamento eficiente, podendo levar essas duas devolutivas para a população, visto que não houve diferença significativa entre esses tratamentos, de acordo com a análise de variância. O balde com água não impactou a ponto de tornar a umidade mais adequada para o bem-estar populacional.

Os recursos ideais seriam o umidificador ou a toalha molhada. No entanto, existem cuidados básicos a serem adotados em relação ao umidificador. Isso se deve ao acúmulo de umidade e poeira que ocorre em ambientes climatizados, e, portanto, a proliferação de micróbios e bactérias é muito maior do que em ambientes abertos. Medidas cabíveis devem ser adotadas em relação ao umidificador, uma vez que não é recomendado deixá-lo ligado no quarto durante toda a noite, pois, sem a iluminação solar, a umidade do ar já se eleva naturalmente, e o excesso de umidade promovida pelo aparelho torna-se um problema, culminando no aparecimento de mofo e bolor. Dentre os principais sintomas de pessoas ocupantes dos ditos ambientes, destacam-se as infecções, as reações alérgicas e irritantes, as dores de cabeça e articulares, a irritação nos olhos, no nariz e na garganta, a tosse seca, as dermatites, a fadiga, a sonolência, a dificuldade de concentração, a sensibilidade a odores, a congestão, a sinusite, a falta de ar, a rinite alérgica, a asma brônquica, entre outros que aumentam mais ainda nessa época de seca. O ideal seria deixá-lo ligado no quarto de 3 a 4 horas seguidas e desligá-lo antes de deitar. Porém, questiona-se como seria possível a pequena elevação na umidade gerada pelo umidificador causar esses problemas, pois a umidade ainda permanece em nível abaixo do recomendado pela OMS. Outra observação em relação ao uso do umidificador seria a necessidade de lavá-lo a cada uso, a fim de evitar que ele seja foco de fungos e outros microrganismos que serão jogados no ar.^( 8 , 13 - 15 )^

É notória a evidente limitação de estudo sobre quais medidas assistencialistas são realmente eficazes e seus impactos na vida da população. No entanto, por meio do conhecimento prévio e da somatória proporcionada pela coleta de dados de acordo com os higrômetros utilizados, foi possível analisar o que realmente funciona e é eficaz. Identificam-se ainda duas possíveis medidas assistencialistas de muita eficácia para população, que podem viabilizar um melhor direcionamento para a estratégia de educação ambiental e métodos de prevenção para população em épocas de seca e baixa umidade, diminuindo, portanto, o impacto de doenças.

## CONCLUSÃO

Este trabalho apresentou os resultados de uma avaliação comparativa do desempenho de três medidas assistencialistas para promover a umidificação do ambiente interno de uma edificação residencial em época de baixa umidade entre os meses de julho e agosto de 2019 em Goiânia, Goiás na Região Centro-Oeste. O umidificador e a toalha foram os tratamentos considerados mais eficientes, e houve efeito significativo da distância sobre a umidade. A distância mais eficiente no aumento e/ou manutenção da umidade do ar foi a de 1m. Assim, ambientalmente sustentável e economicamente viável, o uso de toalhas molhadas para uma população de classe média baixa pode induzir a melhores noites de sono, mesmo nas condições de baixa umidade e seca no Centro-Oeste brasileiro.
